# *Davallia mariesii* Moore Improves Fc*ε*RI-Mediated Allergic Responses in the Rat Basophilic Leukemia Mast Cell Line RBL-2H3 and Passive Cutaneous Anaphylaxis in Mice

**DOI:** 10.1155/2017/8701650

**Published:** 2017-10-19

**Authors:** Hyun Ju Do, Tae Woo Oh, Ju Hye Yang, Kwang Il Park, Jin Yeul Ma

**Affiliations:** Korean Medicine (KM)-Application Center, Korea Institute of Oriental Medicine (KIOM), 70 Cheomdan-ro, Dong-gu, Daegu 41062, Republic of Korea

## Abstract

*Davallia mariesii* Moore (Drynaria rhizome extract (DRE)) is widely known for its efficacy in treating inflammation, arteriosclerosis, and bone injuries. This study evaluated whether treatment with DRE inhibited Fc*ɛ*RI-mediated allergic responses in the RBL-2H3 mast cells and investigated the early- and late-phase mechanisms by which DRE exerts its antiallergic effects. IgE anti-DNP/DNP-HSA-sensitized RBL-2H3 mast cells were tested for cytotoxicity to DRE, followed by the assessment of *β*-hexosaminidase release. We measured the amounts of inflammatory mediators (e.g., histamine, PGD_2_, TNF-*α*, IL-4, and IL-6) and examined the expression of genes involved in arachidonate and Fc*ε*RI signaling pathways. In addition, we confirmed the antiallergic effects of DRE on passive cutaneous anaphylaxis (PCA) in mice. DRE inhibited RBL-2H3 mast cell degranulation and production of allergic mediators in them. In early allergic responses, DRE reduced expression of Fc*ε*RI signaling-related genes (e.g., Syk, Lyn, and Fyn) and extracellular signal-regulated kinase phosphorylation in mast cells. In late allergic responses, DRE reduced PGD_2_ release and COX-2 expression and cPLA_2_ phosphorylation in Fc*ɛ*RI-mediated mast cells. Lastly, 250–500 mg/kg DRE significantly attenuated the IgE-induced PCA reaction in mice. These findings provide novel information on the molecular mechanisms underlying the antiallergic effects of DRE in Fc*ɛ*RI-mediated allergic responses.

## 1. Introduction

Allergies have become a worldwide clinical health problem, and their incidence is rapidly increasing due to various factors [1]. Allergic responses include itching, anaphylaxis, asthma, rhinitis, conjunctivitis, angioedema, urticaria, eczema, eosinophilic disorders (including eosinophilic esophagitis), and drug allergies [2, 3]. The basic treatment for allergies includes avoiding allergens and taking antihistamines, steroids, or immunosuppressants [4]. Many drugs derived from chemical compounds have been developed to treat allergies, but in some cases, they induce side effects or exhibit low efficacy [5]. More recently, to address these problems, natural compounds that regulate allergic reactions have been studied [6]. *Davallia mariesii* Moore, known as Drynaria rhizome (DR) (“gol-se-bo” in Korean and “gu-sui-bu” in Chinese), is a traditional Korean and Chinese medicine known for its efficacy in treating inflammation, osteoporosis, traumatic brain injury, and arteriosclerosis [7, 8]. It is commonly used for treating orthopedic disorders and has been claimed to have therapeutic effects on bone healing [9]. In addition, a few studies have reported that DR inhibits the development of atopic dermatitis-like lesions in a house dust mite antigen-induced atopic dermatitis animal model [10]. However, the cellular signaling mechanisms underlying its antiallergic effects have not been studied.

Mast cells, which are rich in granules in their cytoplasm, are found in body areas, such as the skin surface, the mucosa of the gastrointestinal tract, serous membranes, and in the vicinity of lymphatic vessels and blood vessels, that come in contact with external stimuli. IgE antibodies, compound 48/80, protein kinase C activator, and calcium ionophores have been reported to induce mast cell degranulation [11, 12]. In particular, the allergen IgE-Fc*ε*RI complex, formed by binding of IgE to Fc*ε*RI and expressed on cellular surfaces, triggers early and late allergic responses and chronically prolongs inflammation by secreting chemical mediators and cytokines [6]. Fc*ε*RI, a receptor expressed on the cell membranes of mast cells, comprises *α* and *β* subunits and two *γ* subunits. The *α* chain is involved in extracellular binding of IgE to antigens, whereas the *β* and *γ* chains mediate intracellular signaling [13]. Histamine derived from histidine decarboxylase is primarily released upon mast cell degranulation, and arachidonic acid is released from phospholipids of the cell membrane by phospholipase A2 (cPLA_2_) [14]. Cyclooxygenase-2 (COX-2) induces the synthesis and secretion of lipid metabolites, such as prostaglandins and leukotrienes, that trigger inflammation and pain [15]. These mediators induce immediate hypersensitivity reactions. Cell signaling begins when Src family kinases phosphorylate the *β* subunits of Fc*ε*RI receptors in mast cells stimulated by an antigen [16]. Lyn phosphorylates the tyrosine-phosphorylated immunoreceptor tyrosine-based activation motif (ITAM) of Fc*ε*R, which is a high-affinity IgE receptor. The binding of Syk to phosphorylated ITAMs is regulated by two SH2 domains; it causes structural changes in Syk, resulting in increased enzymatic activity [17]. Subsequently, other multiple signaling and adaptor molecules, including ERK, PLC*γ*1, and Akt, form Syk-receptor complexes, thereby activating the overall signaling system [18].

In the present study, we hypothesized that DRE influences the phosphorylation of Src family kinases and antiallergic effects, including early- and late-phase reactions, in mast cells, ultimately exerting beneficial effects against IgE-mediated allergies by reducing mast cell degranulation. To test this hypothesis, we examined the effect of DRE supplementation on proinflammatory cytokines, including TNF-*α*, IL-4, and IL-6, in IgE-mediated RBL-2H3 mast cells. Furthermore, we assessed the roles of DRE in the Fc*ε*RI signaling pathway (e.g., on Syk, Lyn, and Fyn) and the arachidonate signaling pathway (e.g., on COX-2 and cPLA_2_) as well as its effects on IgE-mediated allergic responses in RBL-2H3 mast cells.

## 2. Materials and Methods

### 2.1. Chemicals

Rat basophilic leukemia mast cell line RBL-2H3 was obtained from the American Type Culture Collection (Manassas, VA, USA). Minimum essential medium alpha modification (MEM-*α* medium), Dulbecco's phosphate-buffered saline, fetal bovine serum (FBS), and antibiotics (100,000 unit/L penicillamine and 100 mg/L streptomycin) were purchased from GE Healthcare Life Sciences (HyClone™, Logan, UT, USA). Dinitrophenyl human serum albumin (DNP-HSA), DNP-immunoglobulin E (IgE anti-DNP), and dexamethasone were obtained from Sigma-Aldrich (St. Louis, MO, USA). 3-(4,5-Dimethylthiazol-2-yl)-2,5-diphenyltetrazolium bromide (MTT) was purchased from Amresco (Solon, OH, USA). *D. mariesii* Moore (Drynaria rhizome extract (DRE)) was obtained from Korean Medicine Application Center (Daegu, Korea).

### 2.2. Preparation of a Water Extract of Drynaria Rhizome

DR was obtained from Yeongcheon hyundai herbal market (Yeongcheon, Korea) and verified by Professor Ki Hwan Bae, Chungnam National University, Republic of Korea. To prepare the DRE, dried DR (30.0 g) were placed in 1000 mL distilled water and then extracted by 3 h of heating at 115°C (Gyeongseo Extractor Cosmos-600, Incheon, Korea). Following extraction, the solution was filtered using standard testing sieves (150 *μ*m) (Retsch, Haan, Germany). Samples were freeze-dried and stored at −20°C before use. Sample acquisition was 5.2 g, and the yield was 17.5%. The powder of DRE was dissolved in 10% DMSO solution for all experiments.

### 2.3. Cell Culture

Rat RBL-2H3 mast cells were grown in MEM-*α* medium supplemented with 10% heat-inactivated FBS containing 1% antibiotics (ABS). Prior to the experiments, 3 × 10^5^ cells were seeded on a six-well plate and grown to confluence for 24 h. At day 2 post-confluence, the medium was replaced with the MEM-*α* medium (10% FBS and 1% ABS) containing IgE anti-DNP (0.1 *μ*g/mL) for 16 h. The medium was replaced with the serum-free medium (MEM-*α* medium with 1% FBS and 1% ABS) containing DRE (100–500 *μ*g/mL) and dexamethasone (100 nM) for 1 h and then treated with DNP-HSA (0.1 *μ*g/mL) for 4 h and/or 30 min. The cells were maintained at 37°C in a humidified atmosphere of 95% air and 5% CO_2_.

### 2.4. MTT Assay

DRE was prepared in a 10% dimethyl sulfoxide (DMSO) solution at a concentration of 100 mg/mL. RBL-2H3 mast cells were tested with increasing concentrations of DRE for 1 h. Cell viability was analyzed after adding 0.5 mg/mL MTT in each well, followed by incubation for 40 min at 37°C. After removing the medium, the cells were lysed with DMSO. Absorbance at 570 nm wavelength was measured using a microplate reader.

### 2.5. N-Acetyl-*β*-d-glucosaminidase (*β*-Hexosaminidase) Release Assay

The culture medium of IgE-sensitized RBL-2H3 mast cells treated with DRE was mixed with 25 *μ*L of 4-methyl-umbellyferyl-N-acetyl-*β*-d-glucosaminidase (10 mM p-NAG) in sodium citrate buffer (0.1 M, pH 4.5) and then incubated for 1 h at 37°C, according to the method described by Park et al. [6]. The reaction was stopped by adding sodium carbonate buffer (0.1 M, pH 10.0). Absorbance at 405 nm wavelength was measured using a microplate reader.

### 2.6. Measurement of Inflammatory Mediators

Histamine, PGD_2_, TNF-*α*, IL-4, and IL-6 levels in the culture medium of IgE-sensitized RBL-2H3 mast cells treated with DRE were determined using enzyme-linked immunosorbent assay kits, as per the manufacturer's instructions.

### 2.7. Immunoblot Analysis

The cells were scraped from the plates using RIPA Lysis Buffer (Merck Millipore, Darmstadt, Germany) containing a protease and phosphatase inhibitor cocktail (Roche, Basel, Switzerland). After incubation on ice for 30 min, the cell lysates were centrifuged at 14,000 rpm for 20 min at 4°C. Proteins were quantified using BCA protein assay (Thermo Scientific, Waltham, MA, USA). The protein lysates were resolved on 10% sodium dodecyl sulfate polyacrylamide gels and then transferred to a polyvinylidene difluoride membrane. Anti-COX-2, anti-phospho-cPLA_2_, anti-phospho-Lyn, anti-phospho-Syk, anti-phospho-Fyn, anti-phospho-PLC*γ*1, anti-phospho-ERK, anti-phospho-Akt, and anti-*α*-tubulin antibodies (Cell Signaling Technology, Boston, MA, USA) were used to detect COX-2, p-cPLA_2_, p-Lyn, p-Syk, p-Fyn, p-PLC*γ*1, p-ERK, p-Akt, and *α*-tubulin, respectively. *α*-Tubulin was used as protein loading control. Blots were observed using a western blot detection kit (Thermo Scientific, Waltham, MA, USA). Protein bands were quantified using Image Lab software (Bio-Rad Laboratories, Richmond, CA, USA).

### 2.8. Animals

Male ICR mice (*n* = 25; 5 weeks old) were randomly assigned to five groups (all *n* = 5) after 1 week adaptation period: control group (CTL), IgE anti-DNP/DNP-HSA group (IgE anti-DNP/DNP-HSA), IgE anti-DNP/DNP-HSA treated with 10 mg/kg dexamethasone group (Dex), IgE anti-DNP/DNP-HSA treated with 250 mg/kg DRE group (DRE 250), and IgE anti-DNP/DNP-HSA treated with 500 mg/kg DRE group (DRE 500). DRE was prepared in 0.5% low-viscosity carboxymethyl cellulose sodium salt (CMC), and CTL and IgE anti-DNP/DNP-HSA groups received equivalent volumes of vehicle (0.5% CMC). The mice were housed under standard laboratory conditions (21°C–24°C and 40%–60% humidity) and were maintained at a 12 h light/12 h dark cycle (lights on at 8:00), with ad libitum access to food and water. All experiments were approved by the Committee on Animal Experimentation and Ethics of KIOM.

### 2.9. Passive Cutaneous Anaphylaxis (PCA) in Mice

The PCA reaction was evaluated as previously described [6]. IgE anti-DNP (4 *μ*g/mL) was subcutaneously injected into the ears of ICR mice. At day 1, IgE-sensitized mice were administered oral DRE (250 or 500 mg/kg) or dexamethasone (10 mg/kg). One hour later, DNP-HSA (300 *μ*g/mL) containing 1% Evans blue was injected into the tail veins. After 1 h, the mice were anesthetized with CO_2_ and tissues from the treated ears were obtained. The Evans blue dye was removed by the ear tissue, which were then incubated with 1 mL formamide at 63°C for 16 h. The mixtures were centrifuged at 17,000 ×g for 10 min at 4°C. Absorbance at 620 nm wavelength was measured using a microplate reader.

### 2.10. Statistical Analysis

Statistical analysis was performed using GraphPad Prism version 5 (GraphPad Software Inc., San Diego, CA, USA). The results are presented as means ± SE. Differences between the experimental groups were analyzed using one-way analysis of variance with Bonferroni's post hoc testing and *p* < 0.05 as the criterion for significance.

## 3. Results

### 3.1. Effect of DRE on RBL-2H3 Mast Cell Viability

**Figure 1:**
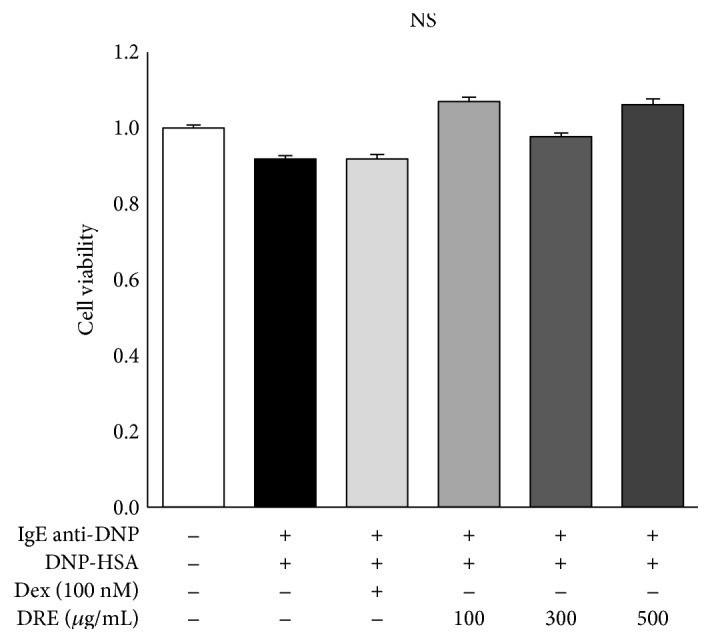
Effects of Drynaria rhizome extract (DRE) on cell viability in RBL-2H3 mast cells. RBL-2H3 mast cell viability was analyzed using an MTT assay after treating the cells with DRE (at 100, 300, and 500 *μ*g/mL) for 1 h and then treating them with DNP-HSA (0.1 *μ*g/mL) for 4 h. The results are expressed as means ± SE calculated from at least three independent experimental results that were tested by analysis of variance with Bonferroni's post hoc testing. NS, not significant at the 0.05 probability level.

To assess cell viability, RBL-2H3 mast cells were treated with DRE at concentrations of 0, 100, 300, and 500 *μ*g/mL (Figure 1). The cell viability was 107% at a concentration of 100 *μ*g/mL and 98% and 106% at concentrations of 300 and 500 *μ*g/mL, respectively.

### 3.2. Effects of DRE on the Inhibition of IgE-Mediated RBL-2H3 Mast Cell Degranulation

**Figure 2:**
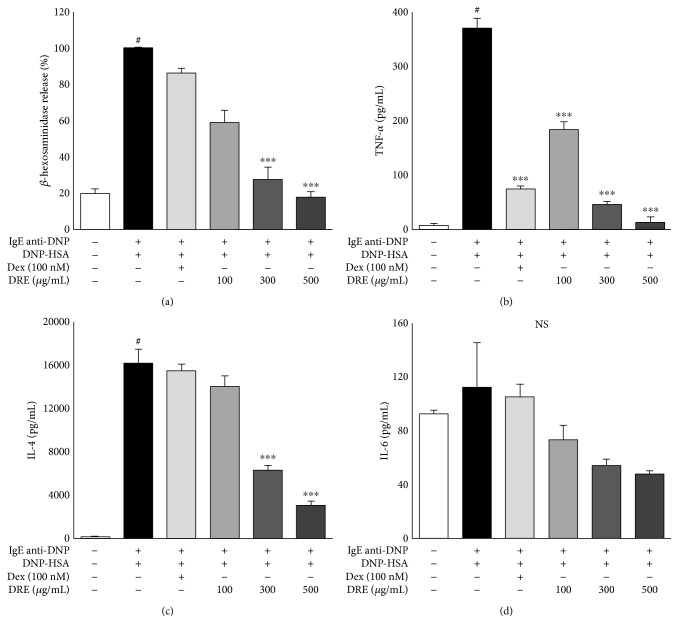
Effect of Drynaria rhizome extract (DRE) at concentrations of 100, 300, and 500 *μ*g/mL on the release of *β*-hexosaminidase and proinflammatory cytokines from RBL-2H3 mast cells. (a) *β*-Hexosaminidase. (b) TNF-*α*. (c) IL-4. (d) IL-6. The results are expressed as the means ± SE of at least three independent experimental results that were tested by analysis of variance with Bonferroni's post hoc testing. ^#^*p* < 0.05, the control group versus the DNP-HSA group; ^∗∗∗^*p* < 0.0005, the DNP-HSA group versus the DRE and Dex treatment group. NS, not significant at the 0.05 probability level.

Mast cells release histamine, cytokines, and other mediators because of degranulation induced by allergic reactions [12]. *β*-Hexosaminidase released as a marker of degranulation from mast cells provides a good indicator of the degree of allergic reactions [19]. The effect of DRE on *β*-hexosaminidase release was concentration-dependent, with a 44% decrease at a concentration of 100 *μ*g/mL, a 76% decrease at 300 *μ*g/mL (*p* < 0.0001), and an 84% decrease at 500 *μ*g/mL (*p* < 0.0001) (Figure 2(a)). These results demonstrated that DRE effectively inhibited IgE-mediated allergic reactions by regulating *β*-hexosaminidase release from mast cells.

### 3.3. Effect of DRE on the Release of Inflammatory Cytokines in RBL-2H3 Mast Cells

We measured the levels of the inflammatory cytokines TNF-*α*, IL-4, and IL-6 in IgE-mediated RBL-2H3 mast cells. DRE at all concentrations significantly reduced the levels of TNF-*α* released by mast cells (Figure 2(b)); TNF-*α* levels at DRE concentrations of 100, 300, and 500 *μ*g/mL were 181.59 pg/mL, 46.01 pg/mL, and 13.01 pg/mL, respectively. Similarly, IL-4 levels were substantially lower in the DRE treatment group than in IgE-mediated RBL-2H3 mast cells (Figure 2(c)). IL-6 levels also demonstrated a concentration-dependent reduction across all DRE concentrations (Figure 2(d)).

### 3.4. Effects of DRE on Late-Phase Reactions in IgE-Mediated RBL-2H3 Mast Cells

**Figure 3:**
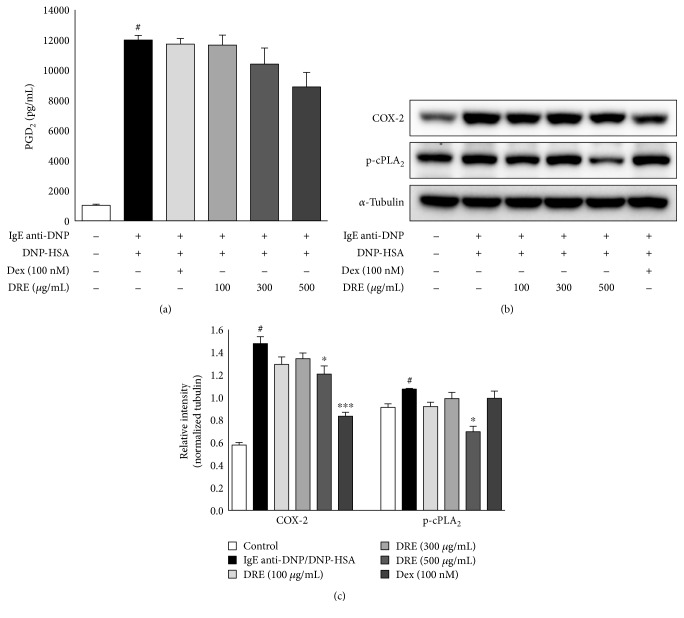
Effect of Drynaria rhizome extract (DRE) on the expression of COX-2 and p-cPLA_2_ genes in RBL-2H3 mast cells. The cells were treated with DRE for 1 h and then treated with DNP-HSA (0.1 *μ*g/mL) for 4 h. (a) PGD_2_ levels in the culture medium of IgE-sensitized RBL-2H3 mast cells treated with DRE. (b) Immunoblot analysis performed with anti-COX-2 and p-cPLA_2_ antibodies. *α*-Tubulin was used as protein loading control. Results are expressed as means ± SE of at least five independent experimental results that were tested by analysis of variance with Bonferroni's post hoc testing; ^#^*p* < 0.05, the control group versus the DNP-HSA group; ^∗^*p* < 0.05 and ^∗∗∗^*p* < 0.0005, the DNP-HSA group versus the DRE and Dex treatment-group.

PGD2 levels demonstrated a tendency to decrease at all concentrations (Figure 3(a); not significant). We measured the effect of DRE on the arachidonate signaling pathway by examining COX-2 and p-cPLA_2_ activation in IgE anti-DNP/DNP-HSA-activated RBL-2H3 mast cells. We observed a decreasing trend in the COX-2 and p-cPLA_2_ protein levels following CRE treatment (Figure 3(b)).

### 3.5. Effects of DRE on Early-Phase Reactions via the Fc*ε*RI Signaling Pathway in RBL-2H3 Mast Cells

**Figure 4:**
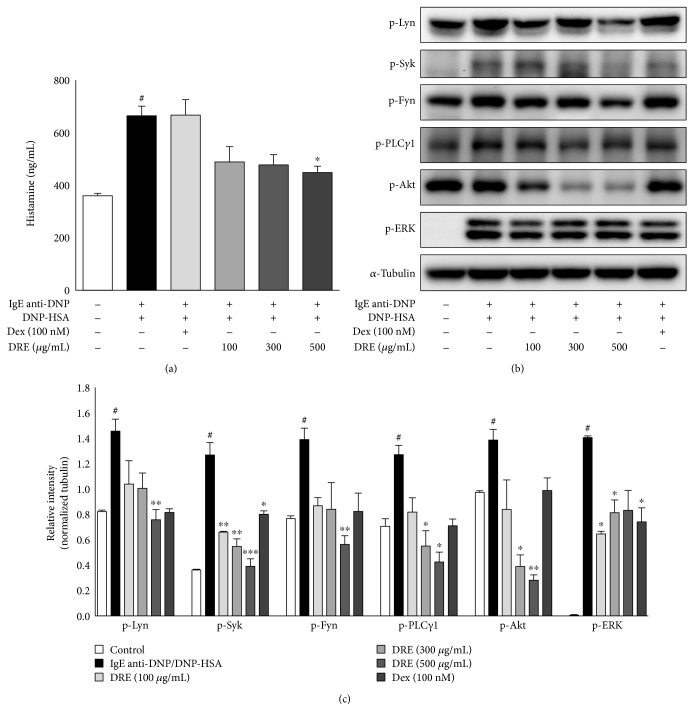
Effect of Drynaria rhizome extract (DRE) on the expression of Src family kinase genes in RBL-2H3 mast cells. The cells were treated with DRE for 1 h and then treated with DNP-HSA (0.1 *μ*g/mL) for 30 min. (a) Histamine levels in the culture medium of IgE-sensitized RBL-2H3 mast cells treated with DRE. (b) Immunoblot analysis performed with anti-p-Syk, anti-p-Lyn, anti-p-Fyn, anti-p-PLC*γ*1, anti-p-ERK, and anti-p-Akt antibodies. *α*-Tubulin was used as protein loading control. Results are expressed as means ± SE of at least five independent experimental results that were tested by analysis of variance with Bonferroni's post-hoc testing; ^#^*p* < 0.05, the control group versus the DNP-HSA group; ^∗^*p* < 0.05, ^∗∗^*p* < 0.005, and ^∗∗∗^*p* < 0.0005, the DNP-HSA group versus the DRE and Dex treatment-group.

We investigated whether DRE influenced the activation of Src tyrosine kinases in IgE anti-DNP/DNP-HSA-activated RBL-2H3 mast cells. We added varying concentrations of DRE to IgE anti-DNP/DNP-HSA-activated RBL-2H3 mast cells and found that DRE inhibited IgE-mediated histamine release from RBL-2H3 mast cells in a concentration-dependent manner (with 26%, 28%, and 33% inhibition at 100, 250, and 500 *μ*g/mL, resp.; Figure 4(a)). We observed a decreasing trend in the levels of the phosphorylated form of Lyn protein following DRE treatment (Figure 4(b)). DRE substantially decreased the levels of the phosphorylated form of Syk protein. However, it had no effect on the levels of the phosphorylated from of Fyn protein. The phosphorylation of ERK, a mitogen-activated protein kinase, was reduced by 100 *μ*g/mL at a concentration of DRE. Akt phosphorylation and PLC*γ*1 phosphorylation were reduced by DRE in a concentration-dependent manner. These results demonstrated that DRE activates the Fc*ε*RI signaling pathway in IgE anti-DNP/DNP-HSA-activated RBL-2H3 mast cells.

### 3.6. Effect of DRE on Allergic Responses in the PCA Model

**Figure 5:**
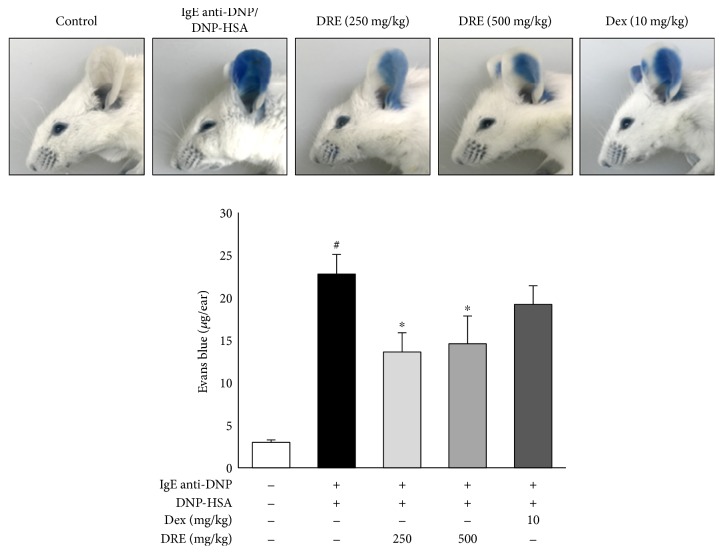
Effect of Drynaria rhizome extract (DRE) on the IgE anti-DNP/DNP-HSA-induced passive cutaneous anaphylaxis model. ICR mice were intradermally administered IgE anti-DNP (4 *μ*g/mL) via the ear for 24 h and were then were orally administered DRE (250 or 500 mg/mL) and dexamethasone (10 mg/mL). One hour later, DNP-HSA (300 *μ*g/mL) containing 1% Evans blue was intravenously injected into their tail veins. After 1 h, the extravasated dye in the ears was analyzed using the procedure described in Materials and Methods. Results are expressed as means ± SE of at least five independent experimental results that were tested by analysis of variance with Bonferroni's post hoc testing; ^#^*p* < 0.05, the control group versus the DNP-HSA group; ^∗^*p* < 0.05, the DNP-HSA group versus the DRE and Dex treatment-group.

We examined the effect of DRE in an animal PCA model representing early-phase allergic responses [6, 20]. The concentration of Evans blue significantly increased from 2.98 ± 0.29 *μ*g/ear in the CTL group to 22.77 ± 2.29 *μ*g/ear in the IgE anti-DNP/DNP-HSA group with the PCA reaction (*p* < 0.0005). Concentrations of Evans blue were significantly lower in the DRE 250 group (13.0 ± 2.28 *μ*g/ear, *p* < 0.05) and the DRE 500 group (14.58 ± 3.28 *μ*g/ear, *p* < 0.05) than in the IgE anti-DNP/DNP-HSA group, but no significant difference was observed between the Dex and IgE anti-DNP/DNP-HSA groups (19.20 ± 2.20 *μ*g/ear) (Figure 5).

## 4. Discussion

Mast cells are the main model for studies on inflammatory and allergic diseases, such as asthma, allergic rhinitis, tissue modification, and rheumatoid arthritis [21]. Their most important features are the presence of a high number of granules within them and the chemical mediators that form crystals. Mast cells express Fc*ε*RI receptors on their cell surface; when antigens cross-link with IgE bound to this receptor, activation begins and degranulation proceeds [22]. It is well known that the rat basophilic leukemia cell line RBL-2H3 plays a role in the mast cell model and is therefore predominantly used to study the IgE-Fc*ε*RI interaction and the signal transduction pathways for the degranulation process [23].

In this study, we found that DRE prevented IgE-induced allergic responses and also observed remarkable reductions in degranulation. Degradation of granules in mast cells or basophils leads to the release of the enzyme *β*-hexosaminidase along with histamine. Therefore, this enzyme is often used as a marker of mast cell degranulation or histamine release [14]. Hexosaminidase assay is used to measure the efficacy of new drugs in preventing mast cell activation and degranulation [19]. Using this assay, we confirmed that DRE inhibited allergen-activated RBL-2H3 mast cell degranulation. Furthermore, DRE influenced allergic reactions, as shown by improvements in the concentrations of inflammatory cytokines. To elucidate the underlying mechanism, we evaluated the control of antiallergic effects and the expression of Fc*ε*RI signaling-related genes known to influence the concentrations of inflammatory cytokines. Histamine released upon mast cell degranulation induces permeability hyperactivity, dilation of blood vessels, secretory action of secretory cells on mucosal surfaces, and a contraction effect on bronchial smooth muscles, leading to an immediate hypersensitivity reaction [24].

The results of the present study also demonstrated that histamine concentration decreased with DRE treatment. In addition, TNF-*α*, IL-4, and IL-6 secretion was inhibited in a concentration-dependent manner. The cytokines TNF-*α*, IL-4, and IL-6 are secreted mainly by T cells; they promote and sustain the inflammatory response, resulting in local and systemic effects in the human body [25, 26]. TNF-*α* is a member of a growing family of peptide mediators and plays a crucial role in the pathogenesis of many acute and chronic inflammatory conditions. It is not only a potent inducer of other inflammatory cytokines, including IL-4 and IL-6, but it is also a self-secretory mediator [25]. IL-6 is involved in the growth and differentiation of T cells and B cells; it contributes to the proliferation of mast cells and is a type of inflammatory cytokine, a powerful mediator of inflammatory processes [27]. IL-4 also modulates the inflammatory response owing to its ability to affect adhesion molecule expression and cytokine production in endothelial cells; it promotes the growth and activation of neutrophils, mast cells, T cells, and eosinophils [26]. These observations suggest that DRE significantly inhibits mast cell degranulation and proinflammatory cytokine release.

One possible mechanism for DRE-induced antiallergic activity may be its effect on the Fc*ε*RI signal cascade. IgE-induced mast cell degranulation is associated with Fc*ε*RI receptor activation, which induces the release of various inflammatory mediators, including TNF-*α*, leukotrienes, and prostaglandins via Lyn/Syk pathway phosphorylation [28]. Thus, Lyn and Syk are important intracellular mediators in early signaling following Fc*ε*RI receptor activation [13]. In the present study, Syk was markedly inhibited by DRE, supporting the notion that it is a primary target of DRE. In turn, Syk activation increases MAP kinase family activation [29]. Further support of this observation was provided by the significant reduction in the phosphorylation of ERK1/2, p38, and Akt, which are downstream effectors of Fc*ε*RI, by DRE [16]. Furthermore, DRE also inhibited COX-2 and cPLA_2_ expression and reduced the levels of the COX-2 product PGD_2_, which is enhanced in activated immune cells, including mast cells. The suppressive effects of DRE on PGD_2_ formation may contribute to its increased antiallergic activity, as PGD_2_ may mediate the inflammation associated with IL-4 [30]. These findings suggest that DRE reduces allergic reactions through the suppression of Fc*ε*RI signal cascade and inhibition of the arachidonate signaling pathway.

We next examined how DRE suppresses IgE-mediated PCA in mice. PCA is characterized by an immediate skin reaction at a localized IgE-mediated allergic response in vivo, typically with increased vascular leakage in the skin that can be assessed by an intravenous injection of Evans blue [20]. In vivo, PCA can be identified based on ear swelling and skin color [6, 20]. Consistent with in vitro findings, DRE successfully reduced allergic inflammatory responses in the PCA-induced mice. This result suggested that DRE inhibits IgE-mediated allergy responses by downregulating mast cell activation.

## 5. Conclusions

DRE has antiallergic effects in both IgE anti-DNP/DNP-HSA-activated RBL-2H3 mast cells and IgE anti-DNP/DNP-HSA-mediated PCA reactions. These antiallergic effects of DRE are because of the inhibition of the degranulation process and the production of allergenic mediators, such as histamine, TNF-*α*, IL-4, IL-6, and PGD_2_, caused by antigen-IgE interaction. DRE may be a useful candidate for antiallergic drugs; because it is a natural product, it may be less toxic than current antiallergic drugs.
